# *Drosophila* RSK Influences the Pace of the Circadian Clock by Negative Regulation of Protein Kinase Shaggy Activity

**DOI:** 10.3389/fnmol.2018.00122

**Published:** 2018-04-13

**Authors:** Katherina Beck, Anna Hovhanyan, Pamela Menegazzi, Charlotte Helfrich-Förster, Thomas Raabe

**Affiliations:** ^1^Institute of Medical Radiation and Cell Research, Biozentrum, University of Würzburg, Würzburg, Germany; ^2^Institute of Neurobiology and Genetics, Biozentrum, University of Würzburg, Würzburg, Germany

**Keywords:** *Drosophila*, circadian clock, Period, Timeless, Shaggy kinase, RSK, Coffin–Lowry syndrome

## Abstract

Endogenous molecular circadian clocks drive daily rhythmic changes at the cellular, physiological, and behavioral level for adaptation to and anticipation of environmental signals. The core molecular system consists of autoregulatory feedback loops, where clock proteins inhibit their own transcription. A complex and not fully understood interplay of regulatory proteins influences activity, localization and stability of clock proteins to set the pace of the clock. This study focuses on the molecular function of Ribosomal S6 Kinase (RSK) in the *Drosophila melanogaster* circadian clock. Mutations in the human *rsk2* gene cause Coffin–Lowry syndrome, which is associated with severe mental disabilities. Knock-out studies with *Drosophila* ortholog *rsk* uncovered functions in synaptic processes, axonal transport and adult behavior including associative learning and circadian activity. However, the molecular targets of RSK remain elusive. Our experiments provide evidence that RSK acts in the key pace maker neurons as a negative regulator of Shaggy (SGG) kinase activity, which in turn determines timely nuclear entry of the clock proteins Period and Timeless to close the negative feedback loop. Phosphorylation of serine 9 in SGG is mediated by the C-terminal kinase domain of RSK, which is in agreement with previous genetic studies of RSK in the circadian clock but argues against the prevailing view that only the N-terminal kinase domain of RSK proteins carries the effector function. Our data provide a mechanistic explanation how RSK influences the molecular clock and imply SGG S9 phosphorylation by RSK and other kinases as a convergence point for diverse cellular and external stimuli.

## Introduction

Cell endogenous circadian clocks control daily oscillations in behavior, physiology, metabolism, and gene expression. Synchronization with the environment is achieved by external cues, most notably light, but also other factors such as temperature, social interactions, and feeding can provide relevant time information (Albrecht, 2012; Crane and Young, 2014; Andreani et al., 2015). The molecular oscillators driving circadian outputs share remarkable similarities among different species. In *Drosophila melanogaster*, the key cellular system for circadian timekeeping of sleep-wake cycles is a group of 150 clock neurons residing in the lateral and dorsal brain, which can be further subdivided into several functional distinct groups (Hermann-Luibl and Helfrich-Förster, 2015; Dubowy and Sehgal, 2017). All clock neurons harbor a molecular network comprised of several interconnected transcriptional-translational feedback loops where transcription factors induce expression of clock genes, which encode for proteins that act as regulators of their own expression. The central feedback loop consists of the transcription factors Clock (CLK) and Cycle (CYC), which bind to promotor sequences of *timeless (tim)* and *period (per)*. CLK/CYC-mediated *per* and *tim* transcription starts during mid-day and peaks in the evening. PER and TIM proteins accumulate in the cytoplasm of clock cells only at night; here they form heterodimers necessary for translocation into the nucleus where they reach maximum levels toward the end of the night. Once in the nucleus, PER inhibits CLK/CYC activity and therefore *per* and *tim* transcription. Multiple and cooperative phosphorylation events control function, stability, and timely localization of PER and TIM (Hardin, 2011; Dubowy and Sehgal, 2017). Briefly, protein kinases Nemo, Doubletime [DBT, corresponding to vertebrate Casein Kinase1 (CK1)], Casein Kinase2 (CK2), Shaggy (SGG, the *Drosophila* ortholog of vertebrate GSK3β) and, at least *in vitro*, MAP kinases p38 (Dusik et al., 2014) and ERK (Ko et al., 2010) phosphorylate PER, whereas TIM is the target of CK2 and SGG (Top et al., 2016)

An additional serine-threonine kinase regulating circadian rhythmicity in *Drosophila* and vertebrates is p90 Ribosomal S6 Kinase (RSK) (Butcher et al., 2004; Akten et al., 2009). The mechanism through which RSK regulates the molecular clock is still unknown. The single *Drosophila* RSK isoform shows similar homology to each of the four RSK proteins (RSK1–4) found in vertebrates. RSK proteins are characterized by a N-terminal and a C-terminal kinase domain (NTKD, CTKD) joined by a linker domain and a binding site for the MAP kinase ERK located at the C-terminus. Based on biochemical studies, a sequential activation model for RSK proteins was proposed. Upon binding to RSK, ERK phosphorylates and thereby activates the CTKD. ERK- and CTKD-mediated phosphorylation of the linker region generates a binding site for another kinase (PDK1), which subsequently activates the NTKD as the effector kinase for substrate phosphorylation (Romeo et al., 2012). In this way, RSK proteins mediate ERK signals, but they can also down-regulate ERK by feed-back inhibition. The model of sequential activation was challenged by the finding that *Drosophila* RSK is functional without catalytic activity of the NTKD in the circadian clock (Tangredi et al., 2012).

The identification of multiple interaction partners linked vertebrate RSK proteins to various cellular processes (Romeo et al., 2012; Lara et al., 2013). Notably, loss of RSK2 function in humans causes Coffin–Lowry syndrome (CLS), a rare X-linked disorder, which is associated amongst others with severe intellectual disabilities (Pereira et al., 2010). Knock-out of *RSK2* in mice uncovered a number of neurophysiological and behavioral phenotypes (Poirier et al., 2007; Pereira et al., 2008; Darcq et al., 2011; Mehmood et al., 2011; Morice et al., 2013; Fischer et al., 2017). Furthermore, elevated RSK activity underlies audiogenic seizure susceptibility in a mouse model for Fragile X-syndrome (Sawicka et al., 2016). In *Drosophila*, RSK acts as a negative regulator of ERK during eye development (Kim et al., 2006) and at the larval neuromuscular junction, where it is involved in anterograde axonal transport, synapse formation, and synaptic transmission (Fischer et al., 2009; Beck et al., 2015). Adult *rsk^-^* mutant flies show defects in olfactory, operant and spatial learning as well as shortened circadian periodicity (Putz et al., 2004; Neuser et al., 2008; Akten et al., 2009; Tangredi et al., 2012). The observed behavioral deficits do not correlate with obvious structural brain abnormalities. In addition, the question about molecular targets of RSK in flies remains open.

One potential convergence point to explain the pleiotropic functions of RSK in *Drosophila* is the GSK3β ortholog SGG. GSK3β/SGG kinases are part of diverse signaling pathways and have multiple substrate proteins (Kaidanovich-Beilin and Woodgett, 2011). In vertebrates, a key feature of GSK3β is negative regulation of kinase activity through phosphorylation of a conserved N-terminal located serine residue (S9) by a variety of kinases including AKT, p70S6 Kinase, PKA and RSK (Kaidanovich-Beilin and Woodgett, 2011). Functional studies in *Drosophila* provided a link between phosphatidylinositol-3-kinase (PI3K)-AKT/Target of Rapamycin (TOR)-p70S6 signaling, SGG-S9 phosphorylation and circadian rhythmicity (Zheng and Sehgal, 2010). SGG phosphorylates Period and Timeless as a major prerequisite for their timely nuclear entry (Ko et al., 2010; Top et al., 2016). Correspondingly, modulation of GSK3β/SGG function by overexpression or inhibition changes the phase of the circadian clock (Martinek et al., 2001; Iitaka et al., 2005).

In this report we wanted to address the question, whether RSK integrates into the molecular circadian clock by regulation of SGG activity. We provide evidence that RSK binds to SGG and phosphorylates S9. Notably, the CTKD but not the NTKD mediates phosphorylation of S9, providing an explanation for previous genetic data (Tangredi et al., 2012) and identifying for the first time an exogenous substrate for the CTKD. Loss of RSK function caused up-regulation of SGG activity in the key pacemaker cells. Genetic interaction studies confirmed the functional relevance of the RSK-SGG interaction to maintain circadian periodicity and to regulate PER expression levels. We suggest that RSK and other kinases determine S9 phosphorylation levels of SGG in clock cells, thereby setting the pace of the molecular circadian oscillator in response to diverse stimuli.

## Materials and Methods

### Fly Stocks and Genetics

Flies were maintained at 25°C on standard cornmeal food in a 12 h light–dark (LD) cycle. *Canton Special* (*CS*) was the control and genetic background in behavior experiments. Other fly stocks used were as follows: the viable X-chromosomal *rsk* deletion *Df(1)ign*^Δ58/1^ (in the following referred to as *rsk^-^*, Putz et al., 2004), *per^01^* (Konopka and Benzer, 1971), *UAS-sgg* (Bloomington *Drosophila* stock center, Bl-5435), *UAS-sgg-RNAi* (P{TRiP.HMS01751}attP40), Bl-38293) and the null allele *sgg^D127^* (*sgg^-^*, Ruel et al., 1993b, kindly provided by Ralf Stanewsky). The *sgg^-^*, *rsk^-^* double mutant was generated by meiotic recombination. Several recombinants were selected on basis of their hemizygous lethality and, when crossed with *rsk^-^* flies, absence of RSK expression on Western blots. The genomic transgene *P[rsk]* and the *UAS-rsk* construct were described previously (Rintelen et al., 2001; Beck et al., 2015). Clock cells were identified by expression of a *UAS-GFP* reporter construct with the *clk^856^-promotor-Gal4* driver line (Gummadova et al., 2009). For immunostaining, RNA isolation or protein extraction, flies were entrained in a 12 h LD cycle for at least 3 days and then collected at the indicated time points.

### Locomotor Activity Recordings and Analysis

Locomotor activity of individual virgin female flies (5–7 days old) was recorded using the *Drosophila* Activity Monitor (DAM) system (TriKinetics, Waltham, MA, United States). During the experiment, a medium of agar and sucrose was provided as a food source. Laser beam crossings were counted as single bouts and counts binned in intervals of 1 min. The monitors were placed in boxes and kept at a constant temperature of 20°C. The light source in the box consisted of white LEDs (Lumitronix LED-Technik GmbH, Jungingen, DE) programmed to reach a maximum light intensity of about 100 lux. For evenly adjusting light intensity within the light boxes, neutral density filters were used (Lee Filters Worldwide, Hampshire, United Kingdom). Data were collected using the DAMSystem 2.1.3 software. All flies were entrained for 7 days to photoperiods with 12 h of light per day (LD 12:12) and subsequently released in constant darkness. Individual fly activity was plotted in actograms using the ImageJ plugin ActogramJ (Schmid et al., 2011). Rhythmicity of individual flies, within the 10 days of recording in DD, was determined using the periodogram analysis tool. In each out of five independent experiments, 15–20 flies were analyzed per genotype.

### Immunohistochemistry

For immunostainings, adult males were directly collected in fixation solution (4% paraformaldehyde in PBS (10 mM Na_2_HPO_4_, 2 mM KH_2_PO_4_, 2.7 mM KCl, 137 mM NaCl) supplemented with 0.1% Triton X-100). Dissected brains were fixed for 30 min at room temperature, then washed in PBT (PBS plus 0.3% Triton X-100, used for all washing steps) before blocking in PBT supplemented with 5% normal goat serum for 2 h. Incubation with the following primary antibodies was done overnight at 4°C: mouse anti-phospho-Y214-SGG (1:400; clone 5G2F12, Papadopoulou et al., 2004), rabbit anti-ITP (1:10000, Hermann et al., 2013), mouse anti-PDF (1:1500, clone C7, Developmental Studies Hybridoma Bank, Iowa City, IA, United States), rabbit anti-PER (1:750, Stanewsky et al., 1997) and chicken anti-GFP (1:750; Millipore, Upstate, Temecula, CA, United States). Secondary antibodies were AlexaFluor 488, Cy3 or Cy5-conjugated and were purchased from Molecular Probes (Eugene, OR, United States) and Dianova (Hamburg, DE). Embedding of brains was done in Vectashield (Vector Laboratories, Burlingame, CA, United States) and confocal images were collected with identical settings for all genotypes with a Leica SP5 microscope (Leica Microsystems, Wetzlar, DE). Image processing was carried out in an identical manner with the ImageJ distribution Fiji (Schindelin et al., 2012).

### RNA Isolation and RT-qPCR Analysis

Entrained flies were collected and immediately frozen in liquid nitrogen. Total RNA was extracted from heads using TRIzol^®^ reagent according to the manufacturer’s instructions (Ambion^®^, Thermo Fisher Scientific, Waltham, MA, United States). First strand cDNA was synthesized from 2 μg of RNA using High-Capacity cDNA Reverse Transcription Kits (Applied Biosystems, Thermo Fisher Scientific). RT-qPCR was done using PowerUp^TM^ SYBR^TM^ Green Master Mix (Applied Biosystems, Thermo Fisher Scientific) on a StepOnePlus^TM^ (Applied Biosystems, Thermo Fisher Scientific) real-time thermal cycler. Reaction mixtures contained 300 nM of oligonucleotides (see Supplementary Table S1). RT-qPCR conditions were 2 min 50°C and 2 min 95°C holding steps, followed by 40 cycles of 15 s 95°C and 1 min at 60°C. Results were expressed as fold change in expression of the treated sample in relation to untreated samples and relative to the reference gene *rp49*. Mean ± SEM was calculated from triplicate experiments from each of the three independent biological samples per genotype.

### Bacterial Protein Expression and Purification

For bacterial expression the N-terminal part of SGG (amino acids 1–289) as a GST fusion protein, an EcoRI fragment derived from a *pAC-sgg-V5-His* construct (Fischer et al., 2016) was cloned into the *pGEX 6P-1* vector (GE Healthcare Life Science, Buckinghamshire, United Kingdom), The resulting construct was used as a template for *in vitro* mutagenesis to replace the TCC triplet encoding serine 9 for GCC (alanine). Primers are listed in Supplementary Table S1. All constructs were verified by sequencing. Expression in *E. coli* BL21 (DE3) and GST protein purification were done as described previously (Dusik et al., 2014). Protein eluates were dialyzed in 20 mM HEPES (pH 7.5) for 48 h.

### Cell Culture and Immunoprecipitation

*Drosophila* Schneider S2 cells were cultured at 25°C in Schneider medium (Biowest, Nuaillé, FR) supplemented with 10% fetal calf serum (FCS), 50 U/ml Penicillin and 50 μg/ml Streptomycin. For transient expression of Myc-epitope tagged RSK in S2 cells, the coding sequence was subcloned from a previously established *pcDNA3-rsk* plasmid (Akten et al., 2009) with XbaI-NotI into the *pAc5.1* vector (Invitrogen, Thermo Fisher Scientific) modified with 6xMyc-epitope sequences. Kinase dead versions for the NTKD (RSK^K231M^) and the CTKD (RSK^K597M^) were generated by *in vitro* mutagenesis (oligonucleotide sequences listed in Supplementary Table S1). The *pAC-sgg-V5-His* construct was described previously (Fischer et al., 2016). For transient expression, 4 × 10^6^ cells were suspended in 1 ml FCS free medium and seeded in 6-well plates. Transfection mixtures containing 100 μl FCS free medium, 2 μg DNA and 8 μl Cellfectin (Invitrogen, Thermo Fisher Scientific) were applied to the wells and incubated for 2 h before addition of 2 ml of full medium. After 24 h, medium was replaced with starvation medium (3% FCS) for 24 h to suppress mitogenic signaling. Re-stimulation of cells with full medium was done for 10 min before cells were harvested in PBS for immunoprecipitation. Cells were re-suspended in 500 μl lysis buffer (25 mM Tris-HCl pH 7.5, 150 mM NaCl, 2 mM EGTA, 2 mM EDTA, 10% Glycerol, 0,5% NP-40) with protease inhibitors (Roche Complete Cocktail and 1 mM AEBSF). Protein complexes were precipitated by overnight incubation with 2 μg anti-Myc antibody (clone 9E10, Santa Cruz Biotech, Dallas, TX, United States) or 10 μg anti His-tag antibody (clone HIS.H8, Thermo Fisher Scientific) bound to 80 μl protein-G-agarose (Roche Diagnostics, Rotkreuz, CH). Samples were washed three times with lysis buffer for Western Blot analysis and additionally with kinase buffer (20 mM HEPES, pH 7.5) for kinase assays.

### Western Blot

Lysates from adult heads homogenized and sonicated in 2x Laemmli, cell lysates or immunoprecipitated protein complexes were separated by SDS-PAGE and transferred to PVDF membranes. Blots were incubated overnight at 4°C with the following antibodies: mouse anti-SGG (1:500; clone 4G-1E, Millipore), mouse anti-SGG (1:500, clone 7G1F2, Papadopoulou et al., 2004), mouse anti-phospho-S9-SGG (1:250; clone 7G2G5, Papadopoulou et al., 2004), mouse anti-phospho-Y214-SGG (1:250; clone 5G2F12, Papadopoulou et al., 2004), rabbit anti-GAPDH (1:500, Novus Biologicals, Littleton, CO, United States), rabbit anti-PER (1:10000, Stanewsky et al., 1997), mouse anti-Myc (1:1000, clone 9E10, Santa Cruz Biotech.), mouse anti-His-tag antibody (1:2000, clone HIS.H8, Thermo Fisher Scientific), mouse anti-α-Tubulin (1:5000, clone NDM1A, Merck, Darmstadt, DE). After incubation with HRP-coupled secondary antibodies, signal detection was done with the ECL Plus detection reagents (GE Healthcare Life Science) and a ChemoCam ECL Imager equipped with a 16bit camera (Intas, Göttingen, DE). Exposure times were adjusted to allow for quantification of signal intensities within the dynamic range of the camera system.

### *In Vitro* Kinase Assay

Kinase reactions were done with non-radioactive ATP. In brief, 15 μg of purified GST::SGG^(1-289)^ or GST::SGG^(1-289)-S9A^ were diluted in 50 μl kinase buffer supplemented with 20 mM MgCl_2_ and 100 μM ATP. After addition of 10 μl of protein G agarose beads complexed with Myc::RSK, Myc::RSK^K231M^ or Myc::RSK^K597M^ proteins, reactions were carried out at 30°C for 30 min and, if indicated, 400U lambda-phosphatase in the provided buffer (New England Biolabs, Ipswich, MA, United States) were added for 90 min at 30°C. Reactions were stopped by adding Laemmli buffer, boiled, separated by SDS-polyacrylamide gel electrophoresis (SDS-PAGE) and analyzed by Western blot using an antibody directed against phosphorylated S9 in SGG (clone 7G2G5, Papadopoulou et al., 2004).

### Data Analysis

Statistical analyses were performed using Mann–Whitney-*U*-test (Origin Pro9.0.0 b45 software) to determine significant differences between genotypes. For multiple testing within one data set, the level of significance *p* ≤ 0.05 was adjusted with the Bonferroni correction factor. All graphs are presented as mean ± SEM, asterisks depict level of statistical significance ^∗∗∗^*p* ≤ 0.001, ^∗∗^*p* ≤ 0.01, and ^∗^*p* ≤ 0.05.

## Results

### RSK Binds to SGG and Phosphorylates Serine 9 in a N-Terminal Kinase Independent Manner

**FIGURE 1:**
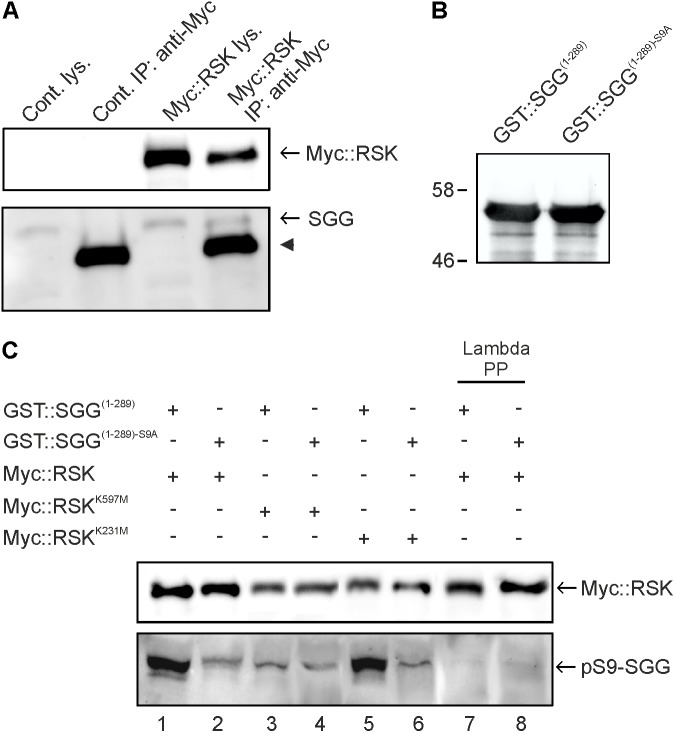
RSK interacts with and phosphorylates SGG at serine 9. **(A)** Lysates from non-transfected Schneider S2 cells (cont) or Myc::RSK transfected cells were directly analyzed by Western blot analysis (lys) or used for immunoprecipitations (IP) with an anti-Myc antibody. The Western blot was probed with anti-Myc and anti-SGG antibodies to detect for co-purification of endogenous SGG. The arrowhead indicates the heavy chain of the anti-Myc antibody from the IP reactions. A representative result from seven independent experiments is shown. **(B)** Input control of bacterially expressed and purified GST::SGG^(1-289)^ or GST::SGG^(1-289)-S9A^ for *in vitro* kinase assays probed with the anti-SGG antibody. Molecular weight markers are indicated. **(C)** RSK C-terminal kinase dependent phosphorylation of SGG at serine 9. Myc::RSK, Myc::RSK^K231M^ and Myc::RSK^K597M^ were immunoprecipitated from transfected S2 cells and used for *in vitro* kinase reactions in the presence of GST::SGG^(1-289)^ or GST::SGG^(1-289)-S9A^ as substrates. Lambda phosphatase treated samples for Myc::RSK in combination with GST::SGG^(1-289)^ or GST::SGG^(1-289)-S9A^ served as controls (lanes 7 and 8). Three independent experiments showed similar results. For uncropped blots, see Supplementary Figure S3.

Biochemical studies in vertebrates identified many interaction partners of RSK proteins, amongst others is protein kinase GSK3β (Kaidanovich-Beilin and Woodgett, 2011). By contrast, direct downstream targets of *Drosophila* RSK remain so far elusive. RSK, like the *Drosophila* GSK3β homolog SGG, is required for axonal transport, synaptic organization, and circadian behavior (Akten et al., 2009; Beck et al., 2015) indicating a direct interaction of both kinases. We confirmed this by transient expression of Myc-tagged RSK (Myc::RSK) in Schneider S2 cells and co-immunoprecipitation of Myc::RSK and endogenous SGG (**Figure 1A**). Visa versa, immunoprecipitation of transiently expressed His-tagged SGG from S2 cells resulted in co-purification of Myc::RSK (Supplementary Figure S1).

In vertebrates, RSK and other kinases phosphorylate GSK3β at serine 9 (S9) (Kaidanovich-Beilin and Woodgett, 2011). Correspondingly, previous studies in *Drosophila* demonstrated phosphorylation of SGG at serine 9 (Papadopoulou et al., 2004). To address the question whether *Drosophila* RSK phosphorylates S9 of SGG, we performed *in vitro* kinase assays using immunoprecipitated Myc::RSK from S2 cells and the bacterially expressed and purified N-terminal part of SGG (GST::SGG^(1-289)^) as a substrate (**Figure 1B**). Since we did not know whether RSK is catalytically active under normal growth conditions, we first starved S2 cells and then stimulated MAP-kinase signaling with full medium before immunoprecipitation of Myc::RSK. Using a phospho-S9 specific antibody (Papadopoulou et al., 2004), phosphorylation of GST::SGG^(1-289)^ was detected (**Figure 1C**, lane 1). Lambda phosphatase treatment resulted in disappearance of the signal, confirming the phosphorylation dependent detection of GST::SGG^(1-289)^ with this antibody (**Figure 1C**, lane 7). To control for specificity of S9 phosphorylation, we introduced a serine to alanine substitution (S9A) into SGG (GST::SGG^(1-289)-S9A^). Using the same amount of GST::SGG^(1-289)-S9A^ as a substrate for Myc::RSK (**Figure 1B**), the phospho-S9 signal was much weaker in comparison to the signal seen with GST::SGG^(1-289)^ (**Figure 1C**, lane 2). The remaining weak signal most likely represents co-purified and phosphorylated endogenous SGG, which has a similar molecular weight as the purified GST::SGG proteins. Disappearance of this signal upon phosphatase treatment supported this conclusion (**Figure 1C**, lanes 7 and 8).

The sequential activation model of RSK postulates that the N-terminal kinase domain (NTKD) mediates substrate phosphorylation upon preceding activation by the C-terminal kinase (CTKD) (Romeo et al., 2012). However, recent findings in *Drosophila* demonstrated that NTKD catalytic activity, but not CTKD activity, is dispensable for normal function of RSK in the circadian clock (Tangredi et al., 2012). To resolve this issue for RSK-mediated SGG phosphorylation, we performed *in vitro* kinase assays with variants of RSK carrying either a kinase inactivating substitution in the NTKD (Myc::RSK^K231M^) or the CTKD (Myc::RSK^K597M^). Whereas Myc::RSK^K231M^ is still able to phosphorylate GST::SGG^(1-289)^ to a similar degree than Myc::RSK (**Figure 1C**, lane 5), Myc::RSK^K597M^ showed no catalytic activity toward GST::SGG^(1-289)^ (**Figure 1C**, lanes 3). Both mutated RSK variants showed no phosphotransferase activity toward GST::SGG^(1-289)-S9A^ (**Figure 1C**, lanes 4 and 6).

This experiment provided for the first time evidence that the CTKD can act independent of NTKD catalytic activity to phosphorylate a substrate protein, at least *in vitro*. It also links the NTKD-independent function of RSK in the circadian clock to SGG as one of the central kinases regulating periodicity (Martinek et al., 2001; Tangredi et al., 2012).

### RSK Negatively Regulates SGG Kinase Activity in Adult Brains

The direct interaction between RSK and SGG raises the question of the functional relationship *in vivo*. Biochemical studies showed that phosphorylation of serine 9 by several kinases blocks GSK3β kinase activity toward substrate proteins, whereas phosphorylation of a conserved tyrosine residue in the kinase activation loop (Y216 in GSK3β, Y214 in SGG) leads to stimulation of kinase activity (Kaidanovich-Beilin and Woodgett, 2011). In *Drosophila*, complexity arises by the expression of several SGG isoforms from a single *sgg* transcription unit, among these SGG39 and SGG10 are the most prominent ones. Based on their ability to rescue the lethality of *sgg^-^* mutant animals, functional redundancy of SGG39 and SGG10 was suggested (Ruel et al., 1993a). Furthermore, only these two isoforms contain the conserved S9 phosphorylation site.

**FIGURE 2:**
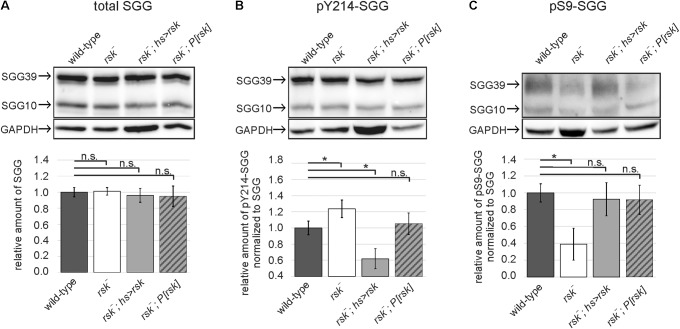
RSK negatively influences SGG activity *in vivo.* Protein lysates from heads of wild-type, RSK loss (*rsk^-^*), RSK overexpression (*rsk^-^; hsP-Gal4; UAS-rsk*) or RSK rescue (*rsk^-^;P[rsk]*) flies were extracted at ZT8 and analyzed for total expression levels of the SGG10 and SGG39 isoforms **(A)**, phospho-Y214-SGG **(B)**, and phospho-S9-SGG **(C)**. Representative Western blots are shown. The bars in the graphs below show the combined band intensity values for SGG10 and SGG39 from five independent biological replicates for all genotypes. Each replicate was independently analyzed. **(A)** To determine relative pan-SGG levels, the house keeping protein GAPDH was used as a reference for normalization and the value for wild-type was set to the arbitrary unit 1. Phospho-Y214-SGG **(B)** and phospho-S9-SGG **(C)** were first normalized to GAPDH and then to the corresponding pan-SGG value for each genotype. Values are presented as relative amount compared to the arbitrary wild-type value 1. For uncropped blots, see Supplementary Figure S4.

To verify a link between RSK, S9 phosphorylation and SGG kinase activity *in vivo*, we first compared overall SGG39 and SGG10 expression levels in adult brains between wild-type, *rsk^-^* mutants, animals overexpressing RSK [heat-shock promotor (*hsP*)-Gal4 driven *UAS-rsk*] and *rsk^-^* mutant flies carrying the genomic rescue construct *P[rsk]*. Quantification of signal intensities normalized to the loading control showed that neither loss nor overexpression of RSK had an influence on overall SGG10 and SGG39 levels (**Figure 2A**). SGG kinase activity levels were monitored with an antibody directed against phosphorylated Y214-SGG (pY214-SGG, Papadopoulou et al., 2004) and normalized to overall SGG levels. *RSK^-^* mutant flies showed a slight but significant increase in Y214 phosphorylation, a phenotype that reverted back to wild-type levels upon expression of the genomic rescue construct (**Figure 2B**). Conversely, *hsP-Gal4* induced expression of the *UAS-rsk* transgene in an *rsk^-^* mutant background resulted in a strong reduction of Y214 phosphorylation (**Figure 2B**). These results indicated that RSK has a negative influence on SGG activity. Based on biochemical evidence this should be mediated by phosphorylation of serine 9. Indeed, up-regulation of SGG activity in *rsk^-^* (**Figure 2B**) correlated with a decreased pS9-SGG signal (**Figure 2C**). Under over-expression conditions in an *rsk^-^* background, pS9-SGG levels raised as expected but did not exceed values of the wild-type or rescue controls (**Figure 2C**). At first sight, this was unexpected because of decreased pY214-SGG levels under over-expression conditions (**Figure 2B**). Since negative regulation is a key feature of SGG/GSK3β proteins (Kaidanovich-Beilin and Woodgett, 2011), S9 phosphorylation levels should be high in order to restrict SGG activation in space and time to specific stimuli. RSK overexpression permanently maintains high S9 phosphorylation, thereby making SGG less responsive for activation, which is reflected by lower pY214 levels (**Figure 2B**).

### A Functional Link Between RSK and SGG to Control Circadian Periodicity

The influence of RSK on SGG activity suggested that the circadian phenotype of *rsk^-^* mutants is caused by de-regulation of SGG. To address this point we performed genetic interaction experiments and immunostainings.

**FIGURE 3:**
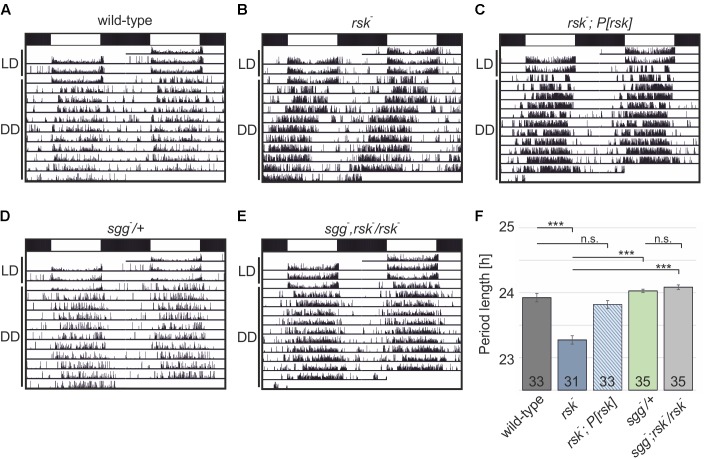
Cooperation of RSK and SGG to regulate circadian periodicity. **(A–E)** Shown are actograms from individual flies of the indicated genotypes trained under 12:12 h light–dark (LD, indicated by white and black bars) before testing them in constant darkness (DD). **(F)** Comparison of period lengths. Data represent the combination of two independent experiments with the number of analyzed flies shown in the bars.

Male flies carrying a deletion of the X-chromosomal *rsk^-^* transcription unit have a shortened circadian period of about 23 h (Akten et al., 2009; Tangredi et al., 2012). Because our analysis required female flies, we first verified the short period phenotype in female *rsk^-^* animals (wild-type: 23.92 ± 0.065, *rsk^-^*: 23.27 ± 0.065, *p* < 0.001) (**Figures 3A,B,F**). Introducing the genomic *rsk* transgene in the *rsk^-^* background reverted the phenotype to wild-type values (*rsk^-^, P[rsk]*: 23.82 ± 0.061, *p* = 0.33 vs. wild-type) (**Figures 3C,F**).

To test for a functional link of RSK and SGG to maintain circadian periodicity, we used the recessive lethal loss-of-function allele *sgg^D127^* (*sgg^-^*). Previous studies showed that elimination of SGG function only in the adult resulted in a pronounced long period phenotype caused by delayed PER/TIM nuclear entry (Martinek et al., 2001). Confirmation came from mutation of relevant SGG phosphorylation sites in TIM and PER (Ko et al., 2010; Top et al., 2016). Heterozygous *sgg^-^*/+ animals showed normal periodicity (*sgg^-^/+*: 24.03 ± 0,026, *p* < 0.001 vs. *rsk^-^* (**Figures 3D,F**). If RSK functions as a negative regulator of SGG kinase activity in the molecular clock, the *rsk^-^* short period phenotype could be caused by enhanced SGG-mediated phosphorylation of PER/TIM, which would promote nuclear entry. If this model is correct, then reducing SGG expression levels by heterozygous *sgg^-^* mutation should counteract enhanced SGG kinase activity in homozygous *rsk^-^* mutants and thereby the short-period phenotype should be at least attenuated. To test this model, X-chromosomal *sgg^-^, rsk^-^* double mutant fly lines were established and crossed with *rsk^-^* flies. Female progeny flies (*sgg^-^, rsk^-^*/*rsk^-^*) showed a periodicity not significantly different from *sgg^-^/+* animals (*sgg^-^, rsk^-^*/*rsk^-^*: 24.09 ± 0.035, *p* < 0.001 vs. *rsk^-^*; *p* = 0.07 vs. *sgg^-^/+*) (**Figures 3B,D–F**) indicating that RSK integrates into the molecular oscillator mainly by negative regulation of SGG activity. Confirmation of this result came from analysis of a second, independent *sgg^-^, rsk^-^* recombinant (data not shown).

Since SGG activity specifically in small (s-)LN_v_ is critical for normal rhythmicity under constant conditions (Top et al., 2016), we focused to this group of clock neurons for further analysis. Clock neurons are subdivided in three groups of dorsal neurons (DN_1-3_) and four groups of lateral neurons [LPN, LN_d_ + 5^th^ LN_v_, large (l-)LN_v_ and s-LN_v_]. These cells express the core clock components, but are functionally distinct with the LN_d_ + 5^th^ LN_v_ and the s-LN_v_ considered as the main circadian pacemaker cells controlling morning and evening activity of the flies (Hermann-Luibl and Helfrich-Förster, 2015; Dubowy and Sehgal, 2017; Helfrich-Förster, 2017; Schubert et al., 2018).

**FIGURE 4:**
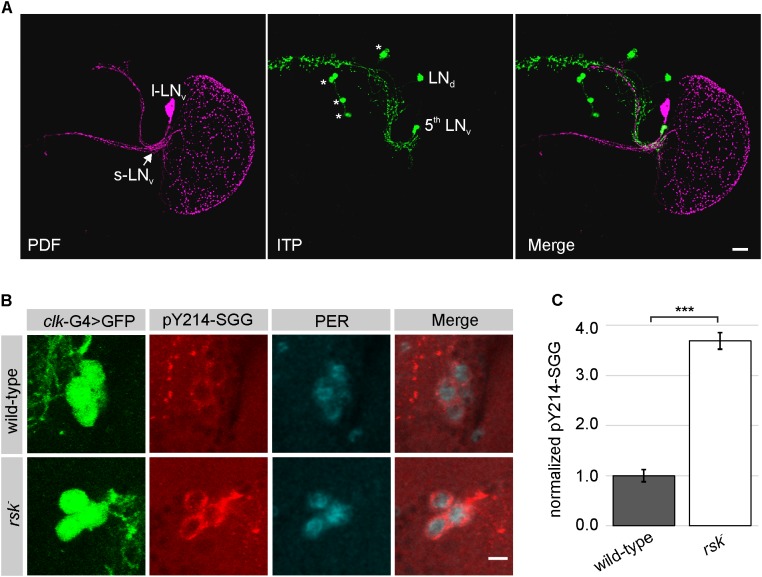
Elevated SGG activity in s-LN_v_ in *rsk^-^* mutants. **(A)** Analysis of the l-LN_v_ and s-LN_v_ arborization patterns in *rsk^-^* by staining for neuropeptide PDF expressed in s-LN_v_ (excluding the 5^th^ LN_v_) and l-LN_v_ (for wild-type see Supplementary Figure S2B) and ITP, which is restricted to the 5^th^ LN_v_ and a single LN_d_ (Hermann-Luibl and Helfrich-Förster, 2015). Asterisks: ITP positive non-clock neurons. Scale bar 20 μm. **(B)** s-LN_v_ in wild-type or *rsk^-^* mutants were identified by position and expression of a GFP-reporter under the control of *clk^856^*-Gal4 driver line. Brains were stained at ZT20 for GFP, PER and activated SGG (pY214-SGG). Pictures are representative examples from at least eight brains analyzed per genotype, recorded and processed in an identical manner. Scale bar: 5 μm. **(C)** Quantification of pY214-SGG signal intensities in s-LN_v_ of *rsk^-^* (*n* = 33 cells in eight brains) compared to wild-type (*n* = 31 cells in eight brains). The pY214-SGG signal in each cell was first normalized to the corresponding GFP-signal and the combined values for wild-type were set to the arbitrary unit 1.

Because of the potential function of RSK in cellular differentiation, we first excluded the possibility that loss of RSK affects the clock neuron network. Stainings against the neuropeptides PDF and ITP verified the presence of l-LN_v_, s-LN_v_, 5^th^ LN_v_ and LN_d_ in *rsk^-^* with no obvious changes in their arborization patterns when compared to wild-type (**Figure 4A**). The initial attempts to directly monitor the influence of RSK on S9 phosphorylation of SGG in s-LN_v_ using the same antibody as for Western blot analysis provided no reliable signals. Therefore, we used the pY214-SGG antibody as an indicator for SGG kinase activity. The time point chosen for analysis was ZT20, when SGG kinase activity should be high to allow PER/TIM phosphorylation and nuclear entry (Shafer et al., 2002). The pY214-SGG antibody labeled the entire brain neuropil and the cytoplasm of the cell bodies, which is consistent with the pleiotropic roles of SGG/GSK3β in different cellular signaling processes, axonal transport and synaptic function (Kaidanovich-Beilin and Woodgett, 2011). In wild-type animals, cytoplasmic pY214-SGG staining in s-LN_v_ was only slightly higher than in the surrounding tissue (**Figure 4B**). By contrast, s-LN_v_ of *rsk^-^* animals showed significantly elevated pY214-SGG staining (**Figures 4B,C**). To verify specificity of the pY214-SGG antibody, we performed *sgg* knock-down and overexpression experiments in clock neurons (Supplementary Figure S2). SGG overexpression resulted in elevated pY214-SGG signals in s-LN_v_ (Supplementary Figure S2A), whereas *sgg* knock-down caused cell swelling, collapse of l-LN_v_ arborizations (Supplementary Figure S2B) and complete disappearance of s-LN_v_. Based on position, few cells could be considered as s-LN_v_, but they were misshaped and either showed no or homogenous PER and residual pY214-SGG staining (Supplementary Figure S2A).

In summary, we propose a function of RSK as a negative regulator of SGG kinase activity in s-LN_v_. The short-period phenotype of *rsk^-^* flies can be explained by enhanced SGG-mediated phosphorylation of PER/TIM resulting in precocious nuclear entry. However, the small differences in circadian period in *rsk^-^* did not allow us to detect changes in the localization profile of PER/TIM by immunohistochemical stainings.

### SGG Mediates the Effect of RSK on *per* Transcription and PER Protein Levels

PER protein undergoes daily oscillations in abundance and phosphorylation with peak levels found at late night (Edery et al., 1994) resulting in feedback repression of CLK/CYC-mediated *per* transcription (Menet et al., 2010). Previous studies demonstrated enhanced PER protein levels in *rsk^-^* animals, which are associated with increased feedback repression of *per* transcription (Akten et al., 2009). These results left open the question whether SGG acts as a mediator for RSK to regulate *per* transcription and PER protein levels.

**FIGURE 5:**
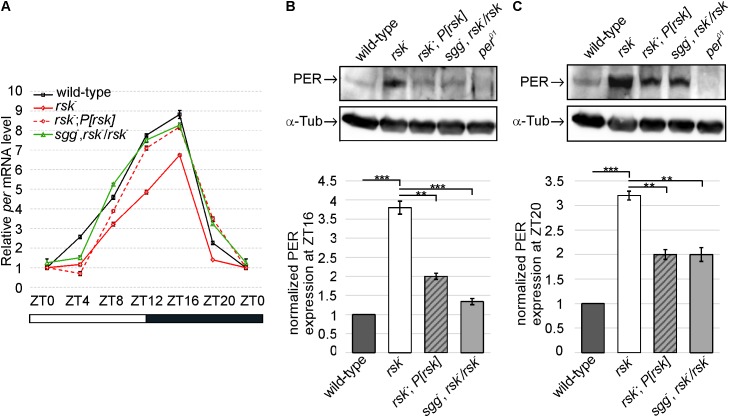
RSK and SGG cooperate to regulate PER-mediated transcriptional feedback repression and PER levels. **(A)** Circadian oscillation of *per* mRNA levels compared between wild-type, *rsk^-^*, *rsk^-^; P[rsk]* and *sgg^-^*, *rsk^-^*/*rsk^-^*. The plot represents the sum of three biological replicates, each repeated in triplicate. *rp49* was used as an internal control to normalize transcript levels. Data points are means ± SEM. **(B,C)** Representative Western blots of adult fly head extracts of the indicated genotypes at ZT 16 **(B)** and ZT20 **(C)** probed for PER and α-Tubulin as a loading control. *per^01^* served as a negative control. Graphs represent measurements of PER levels from four biological replicates normalized to α-Tubulin (mean ± SEM). For uncropped blots, see Supplementary Figure S5.

First, we recapitulated these findings by RT-qPCR analyses of *per* mRNA isolated from head extracts of wild-type and *rsk^-^* flies reared under LD conditions. *per* mRNA levels raise during day and reach peak levels at early night before they drop as a consequence of PER feedback inhibition. Compared to controls, *per* mRNA in *rsk^-^* showed the same periodicity but transcription levels are reduced at most time points (**Figure 5A**). Introducing the genomic *P[rsk]* transgene largely reverted the decrease in *per* mRNA in *rsk^-^* to wild-type values (**Figure 5A**). At the protein level, we analyzed PER expression levels by Western blot at ZT16, when PER accumulates in the cytoplasm thus allowing high CLK/CYC-driven *per* transcription and at ZT20, when nuclear PER mediates feedback repression. At both time points, loss of RSK function resulted in elevated levels of PER compared to wild-type (**Figures 5B,C**). The *P[rsk]* transgene significantly reduced PER levels, but rescue was not complete (**Figures 5B,C**).

If SGG acts as a mediator for RSK to regulate *per* transcription and PER protein levels, reducing the gene dose of *sgg* by half in *sgg^-^, rsk^-^*/*rsk^-^* animals should at least partially suppress excessive accumulation of PER in *rsk^-^* and, as a consequence, increased feedback repression of *per* transcription should be relieved. Indeed, *per* mRNA levels increased close to wild-type values for most time points tested (**Figure 5A**). At the protein level, PER was significantly reduced compared to *rsk^-^* (**Figures 5B,C**). This corresponds to the largely normal transcriptional profile of *per* in *sgg^-^, rsk^-^*/*rsk^-^* animals and correlates with normal circadian behavior (**Figures 3E,F**, **5A**). As a major conclusion we provided evidence that RSK influences feedback repression of *per/tim* transcription by modulating SGG function.

## Discussion

Previous studies showed that clock cells in flies require RSK to perform normal periodicity (Akten et al., 2009), but the molecular mechanism through which the kinase modulates the circadian clock remained elusive. Our biochemical and genetic experiments now provide evidence that RSK negatively regulates kinase activity of the central clock kinase SGG by phosphorylation of serine 9. Notably, we identified SGG as an exogenous substrate of the C-terminal kinase domain of RSK. So far, biochemical studies in vertebrates only verified a function of the C-terminal kinase domain as an intramolecular activator of the N-terminal kinase domain (Romeo et al., 2012). Yet, our finding is in line with previous behavioral studies in flies showing that RSK function in the circadian clock requires a functional C-terminal kinase domain, whereas catalytic activity of the N-terminal kinase domain is dispensable (Tangredi et al., 2012). Notably, during *Drosophila* eye and wing development, RSK acts as a cytoplasmic anchor for ERK and this function is independent of catalytic activity of both kinase domains (Kim et al., 2006). This indicates that the necessity of functional kinase domains in RSK is cellular context dependent.

SGG phosphorylates PER and TIM to finally promote their timely co-translocation to the nucleus (Martinek et al., 2001). Increased SGG activity in *rsk^-^* mutants should promote accumulation of phosphorylated PER/TIM complexes and their accelerated entry into the nucleus, which explains the *rsk^-^* short period phenotype under DD conditions. However, given the pronounced circadian phenotypes under SGG overexpression or loss of function conditions (Martinek et al., 2001), the minor effect of *rsk^-^* on periodicity argues for a modulatory rather than a mandatory role of RSK to control SGG activity. Indeed, also AKT/TOR signaling mediates S9-SGG phosphorylation thereby linking metabolic pathways and circadian behavior (Teleman, 2010; Zheng and Sehgal, 2010). We therefore consider phosphorylation of S9-SGG as a central convergence point for diverse environmental and physiological stimuli to adapt circadian behavior.

Since all clock neurons harbor a molecular oscillator, it is possible that RSK mediated regulation of SGG in maintaining normal free-running rhythm might play a role in more than one clock neuron cluster. Several experiments support the conclusion that the interaction of RSK with SGG in s-LN_v_ plays a central role to maintain periodicity. First, expression of an *rsk* transgene specifically in PDF-expressing neurons (s-LN_v_ and l-LN_v_) rescued the *rsk^-^* short period phenotype (Akten et al., 2009). Second, overexpression of SGG in s-LN_v_, but not in l-LN_v_ caused a short period phenotype indicating that regulation of SGG activity in s-LNs is essential for normal periodicity (Top et al., 2016). The enhanced SGG activity in s-LN_v_ in *rsk^-^* supports this conclusion (**Figure 4**). However, the Western blot analysis from whole brain lysates indicated a global up-regulation of SGG activity in *rsk^-^* (**Figure 2**), which is consistent with the pleiotropic functions of RSK in ERK signaling (Romeo et al., 2012) and of SGG/GSK3β in the Wingless/Wnt pathway (Kaidanovich-Beilin and Woodgett, 2011). Although molecular oscillators act cell-autonomously, recent evidence suggestedcomplex cross-talks and feedback-loops among clock neurons to maintain periodicity (Dissel et al., 2014). Also glia cells integrate into the circadian network (Jackson et al., 2015). Thus a rigorous test for a functional link between RSK and SGG solely in s-LNv to maintain normal periodicity is necessary. However, the observed cell damages upon *sgg* knock-down impede such an analysis (Supplementary Figure S2B).

Could there be alternative routes how RSK acts on SGG to influence PER or TIM? SGG-mediated phosphorylation of PER at serine 657 residue requires preceding phosphorylation of the neighboring serine 661 residue. The MAP-kinases ERK and p38 promote serine 661 phosphorylation (Ko et al., 2010; Dusik et al., 2014) and thus RSK as a downstream effector of ERK might be the relevant kinase. We exclude a function of RSK as a serine 661phosphorylating kinase for two reasons. First, *in vitro* biochemical data suggested direct phosphorylation by ERK or p38 (Ko et al., 2010; Dusik et al., 2014). Second, abrogation of serine 657 or 661 phosphorylation by alanine substitutions resulted in delayed nuclear entry of PER, period lengthening and decreased feedback repression of *per* transcription (Ko et al., 2010). By contrary, loss of RSK causes shortened periodicity and increased feedback repression of *per* transcription, which is not consistent with a function of RSK as a S661 phosphorylating kinase. However, RSK not only acts as an effector of ERK, but negatively regulates ERK activity by feedback inhibition (Romeo et al., 2012). Future studies have to clarify the issue, whether fine tuning of ERK activity by RSK in clock neurons is an additional mechanism.

**FIGURE 6:**
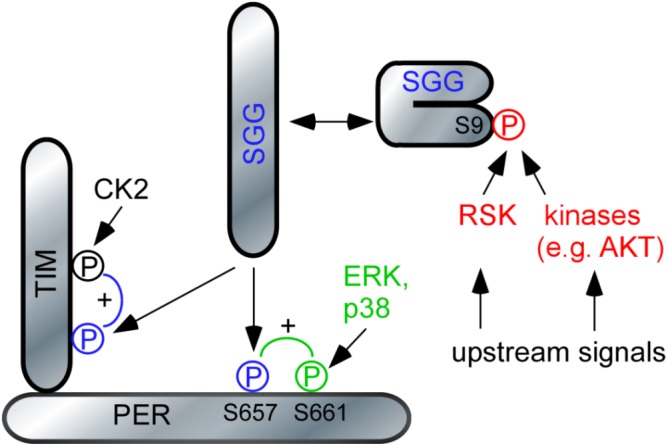
Model of RSK function in the circadian clock. The model relies on previous data demonstrating the hierarchical action of MAP-kinases and SGG on PER (Ko et al., 2010; Dusik et al., 2014) respectively SGG and CK2 on TIM (Top et al., 2016). For simplicity, DBT, CK2 and Nemo-mediated phosphorylation of PER was omitted. SGG is kept in an inactive conformation by phosphorylation of S9. Release of this inhibition promotes nuclear entry of PER/TIM complexes and feedback transcriptional repression. For details, see section “Discussion.”

Our results also help to resolve the conundrum about the relationship between RSK and kinase CK2 in clock cells. CK2 is a heterotetrameric kinase expressed in all eukaryotic cells with a vast array of substrates (Filhol and Cochet, 2009). Mutations in the *Drosophila* CK2 subunits result in period lengthening due to impaired PER/TIM phosphorylation and delayed nuclear entry (Lin et al., 2002; Akten et al., 2003). Double mutant combinations of the “short period” *rsk^-^* mutant and the “long period” *CK2* mutants showed a similar long periodicity than the single *CK2* mutants suggesting a model where RSK negatively regulates CK2 activity (Akten et al., 2009). However, such a model is difficult to reconcile with biochemical and structural data implicating CK2 as a kinase that is not regulated by second messengers (Schnitzler et al., 2014). Based on a recent study showing that CK2-mediated phosphorylation of TIM requires preceding phosphorylation of TIM by SGG (Top et al., 2016), we can now reconcile the conflicting genetic and biochemical data. RSK is a negative regulator of SGG activity, which is required for CK2-mediated phosphorylation of TIM and nuclear entry (**Figure 6**). The model predicts that in *rsk^-^* mutants, elevated SGG activity promotes CK2-mediated phosphorylation of TIM and advanced nuclear entry. In *rsk^-^ CK2* double mutants, CK2-mediated phosphorylation of TIM is impaired. Therefore, these flies have a similar long period phenotype than the *CK2* single mutants, despite enhanced SGG activity caused by lack of RSK.

In summary we propose that RSK has effects on PER and TIM. The hierarchical cascade of RSK-SGG-CK2 controls TIM-dependent nuclear entry of TIM/PER complexes (Jang et al., 2015), whereas RSK-SGG influence the transcriptional repressive function of PER. Since regulation of SGG activity by RSK and other kinases is at the center of both processes, defining the upstream signals for these kinases will provide important information how changes in environmental or physiological condition are translated into appropriate circadian behavioral responses. Furthermore, RSK in *Drosophila* and RSK2 in vertebrates influence a variety of behavioral responses and regulate synaptic function. It will be interesting to investigate a putative RSK-SGG/GSK3β link in a broader neuronal context. With this knowledge our understanding of the pathophysiology of Coffin–Lowry syndrome can be improved.

## Ethics Statement

All *Drosophila* experiments were performed according to animal protection guidelines of the government of Unterfranken, State of Bavaria.

## Author Contributions

TR, KB, AH, and PM designed the the research. KB, AH, CH-F, and TR performed the research. KB, AH, and PM analyzed the data. KB, AH, CH-F, and TR prepared the figures. TR wrote the manuscript with support of all other authors.

## Conflict of Interest Statement

The authors declare that the research was conducted in the absence of any commercial or financial relationships that could be construed as a potential conflict of interest.

## References

[B1] Akten B. Jauch E. Genova G. K. Kim E. Y. Edery I. Raabe T. (2003). A role for CK2 in the *Drosophila* circadian oscillator. *Nat. Neurosci.* 6 251–257. 10.1038/nn1007nn1007 12563262

[B2] Akten B. Tangredi M. M. Jauch E. Roberts M. A. Ng F. Raabe T. (2009). Ribosomal s6 kinase cooperates with casein kinase 2 to modulate the *Drosophila* circadian molecular oscillator. *J. Neurosci.* 29 466–475. 10.1523/JNEUROSCI.4034-08.2009 19144847 PMC2775553

[B3] Albrecht U. (2012). Timing to perfection: the biology of central and peripheral circadian clocks. *Neuron* 74 246–260. 10.1016/j.neuron.2012.04.006 22542179

[B4] Andreani T. S. Itoh T. Q. Yildirim E. Hwangbo D. S. Allada R. (2015). Genetics of circadian rhythms. *Sleep Med. Clin.* 10 413–421. 10.1016/j.jsmc.2015.08.007 26568119 PMC4758938

[B5] Beck K. Ehmann N. Andlauer T. F. Ljaschenko D. Strecker K. Fischer M. (2015). Loss of the Coffin-Lowry syndrome-associated gene *RSK2* alters ERK activity, synaptic function and axonal transport in *Drosophila* motoneurons. *Dis. Models Mech.* 8 1389–1400. 10.1242/dmm.021246 26398944 PMC4631788

[B6] Butcher G. Q. Lee B. Hsieh F. Obrietan K. (2004). Light- and clock-dependent regulation of ribosomal S6 kinase activity in the suprachiasmatic nucleus. *Eur. J. Neurosci.* 19 907–915. 10.1111/j.0953-816X.2004.03155.x 15009138

[B7] Crane B. R. Young M. W. (2014). Interactive features of proteins composing eukaryotic circadian clocks. *Annu. Rev. Biochem.* 83 191–219. 10.1146/annurev-biochem-060713-035644 24905781

[B8] Darcq E. Koebel P. Del Boca C. Pannetier S. Kirstetter A. S. Garnier J. M. (2011). RSK2 signaling in brain habenula contributes to place aversion learning. *Learn. Mem.* 18 574–578. 10.1101/lm.2221011 21852432 PMC3256568

[B9] Dissel S. Hansen C. N. Ozkaya O. Hemsley M. Kyriacou C. P. Rosato E. (2014). The logic of circadian organization in *Drosophila*. *Curr. Biol.* 24 2257–2266. 10.1016/j.cub.2014.08.023 25220056 PMC4188814

[B10] Dubowy C. Sehgal A. (2017). Circadian rhythms and sleep in *Drosophila melanogaster*. *Genetics* 205 1373–1397. 10.1534/genetics.115.185157 28360128 PMC5378101

[B11] Dusik V. Senthilan P. R. Mentzel B. Hartlieb H. Wülbeck C. Yoshii T. (2014). The MAP kinase p38 is part of *Drosophila melanogaster’s* circadian clock. *PLoS Genet.* 10:e1004565. 10.1371/journal.pgen.1004565 25144774 PMC4140665

[B12] Edery I. Zwiebel L. J. Dembinska M. E. Rosbash M. (1994). Temporal phosphorylation of the *Drosophila* period protein. *Proc. Natl. Acad. Sci. U.S.A.* 91 2260–2264. 10.1073/pnas.91.6.2260 8134384 PMC43350

[B13] Filhol O. Cochet C. (2009). Protein kinase CK2 in health and disease: cellular functions of protein kinase CK2: a dynamic affair. *Cell Mol. Life Sci.* 66 1830–1839. 10.1007/s00018-009-9151-1 19387551 PMC11115791

[B14] Fischer M. Cabello V. Popp S. Krackow S. Hommers L. Deckert J. (2017). Rsk2 knockout affects emotional behavior in the IntelliCage. *Behav. Genet.* 47 434–448. 10.1007/s10519-017-9853-3 28585192

[B15] Fischer M. Raabe T. Heisenberg M. Sendtner M. (2009). *Drosophila* RSK negatively regulates bouton number at the neuromuscular junction. *Dev. Neurobiol.* 69 212–220. 10.1002/dneu.20700 19160443

[B16] Fischer R. Helfrich-Förster C. Peschel N. (2016). GSK-3beta does not stabilize Cryptochrome in the circadian clock of *Drosophila*. *PLoS One* 11:e0146571. 10.1371/journal.pone.0146571 26741981 PMC4704813

[B17] Gummadova J. O. Coutts G. A. Glossop N. R. (2009). Analysis of the *Drosophila* Clock promoter reveals heterogeneity in expression between subgroups of central oscillator cells and identifies a novel enhancer region. *J. Biol. Rhythms* 24 353–367. 10.1177/0748730409343890 19755581

[B18] Hardin P. E. (2011). Molecular genetic analysis of circadian timekeeping in *Drosophila*. *Adv. Genet.* 74 141–173. 21924977 10.1016/B978-0-12-387690-4.00005-2 PMC4108082

[B19] Helfrich-Förster C. (2017). “The *Drosophila* clock system,” in *Biological Timekeeping: Clocks, Rhythms and Behaviour*, ed. Kumar V. (Berlin: Springer).

[B20] Hermann C. Saccon R. Senthilan P. R. Domnik L. Dircksen H. Yoshii T. (2013). The circadian clock network in the brain of different *Drosophila* species. *J. Comp. Neurol.* 521 367–388. 10.1002/cne.23178 22736465

[B21] Hermann-Luibl C. Helfrich-Förster C. (2015). Clock network in *Drosophila*. *Curr. Opin. Insect Sci.* 7 65–70. 10.1016/j.cois.2014.11.003 32846682

[B22] Iitaka C. Miyazaki K. Akaike T. Ishida N. (2005). A role for glycogen synthase kinase-3beta in the mammalian circadian clock. *J. Biol. Chem.* 280 29397–29402. 10.1074/jbc.M503526200 15972822

[B23] Jackson F. R. Ng F. S. Sengupta S. You S. Huang Y. (2015). Glial cell regulation of rhythmic behavior. *Methods Enzymol.* 552 45–73. 10.1016/bs.mie.2014.10.016 25707272 PMC4662800

[B24] Jang A. R. Moravcevic K. Saez L. Young M. W. Sehgal A. (2015). *Drosophila* TIM binds importin alpha1, and acts as an adapter to transport PER to the nucleus. *PLoS Genet.* 11:e1004974. 10.1371/journal.pgen.1004974 25674790 PMC4335507

[B25] Kaidanovich-Beilin O. Woodgett J. R. (2011). GSK-3: functional insights from cell biology and animal models. *Front. Mol. Neurosci.* 4:40. 10.3389/fnmol.2011.00040 22110425 PMC3217193

[B26] Kim M. Lee J. H. Koh H. Lee S. Y. Jang C. Chung C. J. (2006). Inhibition of ERK-MAP kinase signaling by RSK during *Drosophila* development. *EMBO J.* 25 3056–3067. 10.1038/sj.emboj.7601180 16763554 PMC1500987

[B27] Ko H. W. Kim E. Y. Chiu J. Vanselow J. T. Kramer A. Edery I. (2010). A hierarchical phosphorylation cascade that regulates the timing of PERIOD nuclear entry reveals novel roles for proline-directed kinases and GSK-3beta/SGG in circadian clocks. *J. Neurosci.* 30 12664–12675. 10.1523/JNEUROSCI.1586-10.2010 20861372 PMC2957474

[B28] Konopka R. J. Benzer S. (1971). Clock mutants of *Drosophila melanogaster*. *Proc. Natl. Acad. Sci. U.S.A.* 68 2112–2116. 10.1073/pnas.68.9.2112 5002428 PMC389363

[B29] Lara R. Seckl M. J. Pardo O. E. (2013). The p90 RSK family members: common functions and isoform specificity. *Cancer Res.* 73 5301–5308. 10.1158/0008-5472.CAN-12-4448 23970478

[B30] Lin J. M. Kilman V. L. Keegan K. Paddock B. Emery-Le M. Rosbash M. (2002). A role for casein kinase 2alpha in the *Drosophila* circadian clock. *Nature* 420 816–820. 10.1038/nature01235 12447397

[B31] Martinek S. Inonog S. Manoukian A. S. Young M. W. (2001). A role for the segment polarity gene *shaggy*/GSK-3 in the *Drosophila* circadian clock. *Cell* 105 769–779. 10.1016/S0092-8674(01)00383-X 11440719

[B32] Mehmood T. Schneider A. Sibille J. Marques Pereira P. Pannetier S. Ammar M. R. (2011). Transcriptome profile reveals AMPA receptor dysfunction in the hippocampus of the Rsk2-knockout mice, an animal model of Coffin-Lowry syndrome. *Hum. Genet.* 129 255–269. 10.1007/s00439-010-0918-0 21116650

[B33] Menet J. S. Abruzzi K. C. Desrochers J. Rodriguez J. Rosbash M. (2010). Dynamic PER repression mechanisms in the *Drosophila* circadian clock: from on-DNA to off-DNA. *Genes Dev.* 24 358–367. 10.1101/gad.1883910 20159956 PMC2816735

[B34] Morice E. Farley S. Poirier R. Dallerac G. Chagneau C. Pannetier S. (2013). Defective synaptic transmission and structure in the dentate gyrus and selective fear memory impairment in the Rsk2 mutant mouse model of Coffin-Lowry syndrome. *Neurobiol. Dis.* 58 156–168. 10.1016/j.nbd.2013.05.016 23742761

[B35] Neuser K. Triphan T. Mronz M. Poeck B. Strauss R. (2008). Analysis of a spatial orientation memory in *Drosophila*. *Nature* 453 1244–1247. 10.1038/nature07003 18509336

[B36] Papadopoulou D. Bianchi M. W. Bourouis M. (2004). Functional studies of shaggy/glycogen synthase kinase 3 phosphorylation sites in *Drosophila melanogaster*. *Mol. Cell. Biol.* 24 4909–4919. 10.1128/MCB.24.11.4909-4919.2004 15143183 PMC416399

[B37] Pereira P. M. Gruss M. Braun K. Foos N. Pannetier S. Hanauer A. (2008). Dopaminergic system dysregulation in the mrsk2_KO mouse, an animal model of the Coffin-Lowry syndrome. *J. Neurochem.* 107 1325–1334. 10.1111/j.1471-4159.2008.05703.x 18823370

[B38] Pereira P. M. Schneider A. Pannetier S. Heron D. Hanauer A. (2010). Coffin-Lowry syndrome. *Eur. J. Hum. Genet.* 18 627–633. 10.1038/ejhg.2009.189 19888300 PMC2987346

[B39] Poirier R. Jacquot S. Vaillend C. Soutthiphong A. A. Libbey M. Davis S. (2007). Deletion of the Coffin-Lowry syndrome gene Rsk2 in mice is associated with impaired spatial learning and reduced control of exploratory behavior. *Behav. Genet.* 37 31–50. 10.1007/s10519-006-9116-1 17033934

[B40] Putz G. Bertolucci F. Raabe T. Zars T. Heisenberg M. (2004). The S6KII (rsk) gene of *Drosophila melanogaster* differentially affects an operant and a classical learning task. *J. Neurosci.* 24 9745–9751. 10.1523/JNEUROSCI.3211-04.2004 15525759 PMC6730233

[B41] Rintelen F. Stocker H. Thomas G. Hafen E. (2001). PDK1 regulates growth through Akt and S6K in *Drosophila*. *Proc. Natl. Acad. Sci. U.S.A.* 98 15020–15025. 10.1073/pnas.011318098 11752451 PMC64976

[B42] Romeo Y. Zhang X. Roux P. P. (2012). Regulation and function of the RSK family of protein kinases. *Biochem. J.* 441 553–569. 10.1042/BJ20110289 22187936

[B43] Ruel L. Bourouis M. Heitzler P. Pantesco V. Simpson P. (1993a). *Drosophila shaggy* kinase and rat glycogen synthase kinase-3 have conserved activities and act downstream of *Notch*. *Nature* 362 557–560. 10.1038/362557a0 8385271

[B44] Ruel L. Pantesco V. Lutz Y. Simpson P. Bourouis M. (1993b). Functional significance of a family of protein kinases encoded at the shaggy locus in *Drosophila*. *EMBO J.* 12 1657–1669. 8467811 10.1002/j.1460-2075.1993.tb05811.x PMC413380

[B45] Sawicka K. Pyronneau A. Chao M. Bennett M. V. Zukin R. S. (2016). Elevated ERK/p90 ribosomal S6 kinase activity underlies audiogenic seizure susceptibility in fragile X mice. *Proc. Natl. Acad. Sci. U.S.A.* 113 E6290–E6297. 10.1073/pnas.1610812113 27663742 PMC5068319

[B46] Schindelin J. Arganda-Carreras I. Frise E. Kaynig V. Longair M. Pietzsch T. (2012). Fiji: an open-source platform for biological-image analysis. *Nat. Methods* 9 676–682. 10.1038/nmeth.2019 22743772 PMC3855844

[B47] Schmid B. Helfrich-Förster C. Yoshii T. (2011). A new ImageJ plug-in “ActogramJ” for chronobiological analyses. *J. Biol. Rhythms* 26 464–467. 10.1177/0748730411414264 21921300

[B48] Schnitzler A. Olsen B. B. Issinger O. G. Niefind K. (2014). The protein kinase CK2(Andante) holoenzyme structure supports proposed models of autoregulation and trans-autophosphorylation. *J. Mol. Biol.* 426 1871–1882. 10.1016/j.jmb.2014.02.018 24594356

[B49] Schubert F. K. Hagedorn N. Yoshii T. Helfrich-Förster C. Rieger D. (2018). Neuroanatomical details of the lateral neurons of *Drosophila melanogaster* support their functional role in the circadian system. *J. Comp. Neurol.* 526 1209–1231. 10.1002/cne.24406 29424420 PMC5873451

[B50] Shafer O. T. Rosbash M. Truman J. W. (2002). Sequential nuclear accumulation of the clock proteins period and timeless in the pacemaker neurons of *Drosophila melanogaster*. *J. Neurosci.* 22 5946–5954. 12122057 10.1523/JNEUROSCI.22-14-05946.2002 PMC6757919

[B51] Stanewsky R. Frisch B. Brandes C. Hamblen-Coyle M. J. Rosbash M. Hall J. C. (1997). Temporal and spatial expression patterns of transgenes containing increasing amounts of the *Drosophila* clock gene period and a *lacZ* reporter: mapping elements of the PER protein involved in circadian cycling. *J. Neurosci.* 17 676–696. 8987790 10.1523/JNEUROSCI.17-02-00676.1997 PMC6573240

[B52] Tangredi M. M. Ng F. S. Jackson F. R. (2012). The C-terminal kinase and ERK-binding domains of *Drosophila* S6KII (RSK) are required for phosphorylation of the protein and modulation of circadian behavior. *J. Biol. Chem.* 287 16748–16758. 10.1074/jbc.M111.315929 22447936 PMC3351340

[B53] Teleman A. A. (2010). Molecular mechanisms of metabolic regulation by insulin in *Drosophila*. *Biochem. J.* 425 13–26. 10.1042/BJ20091181 20001959

[B54] Top D. Harms E. Syed S. Adams E. L. Saez L. (2016). GSK-3 and CK2 kinases converge on timeless to regulate the master clock. *Cell Rep.* 16 1–11. 10.1016/j.celrep.2016.06.005 27346344 PMC4945451

[B55] Zheng X. Sehgal A. (2010). AKT and TOR signaling set the pace of the circadian pacemaker. *Curr. Biol.* 20 1203–1208. 10.1016/j.cub.2010.05.027 20619819 PMC3165196

