# Supplementary material for: Drosophila RSK Influences the Pace of the Circadian Clock by Negative Regulation of Protein Kinase Shaggy Activity
Source: Front Mol Neurosci. 2018 Apr 13;11:122. doi: 10.3389/fnmol.2018.00122 (PMC5908959; doi:10.3389/fnmol.2018.00122)
Supplement: Supplementary file 1 [file Table_1.PDF]

Supplementary table S1

Oligonucleotides for mutagenesis

	**Sequence 5´-3´**
RSK-K231M For.L	GCCATGATGGTGCTCAAAAAGGCCACCCTAAAAGTAAAAG
RSK-K231M For.S	AAGGCCACCCTAAAAGTAAAAG
RSK-K231M Rev.L	TTGAGCACCATCATGGCATAGAGTGTTCCTGCATCTTTGC
RSK-K231M Rev.S	ATAGAGTGTTCCTGCATCTTTGC
RSK-K597M For.L	CGCAGTAATGGTAATCGAAAAGGCAGCTGTGGCCG
RSK-K597M For.S	GAAAAGGCAGCTGTGGCCG
RSK-K597M Rev.L	GATTACCATTACTGCGTAATGTTTCTTGGAGGCTCGATGC
RSK-K597M Rev.S	TAATGTTTCTTGGAGGCTCGATGC
SGG-S9A For.	CAAGAACTTCCGCCTTCGCCGAG
SGG-S9A Rev.	CTCGGCGAAGGCGGAAGTTCTTG

Oligonucleotides for RT-qPCR

	**Sequence 5´-3´**
Period forward	TAGTAGCCACACCCGCAGT
Period reverse	GGAATGGAAGGGGGAGTTAG
Rp49 forward	GCCCAAGATCGTGAAGAAGC
Rp49 reverse	CGACGCACTCTGTTGTCG
