# Supplementary material for: Drosophila RSK Influences the Pace of the Circadian Clock by Negative Regulation of Protein Kinase Shaggy Activity
Source: Front Mol Neurosci. 2018 Apr 13;11:122. doi: 10.3389/fnmol.2018.00122 (PMC5908959; doi:10.3389/fnmol.2018.00122)
Supplement: Supplementary file 2 [file Data_Sheet_1.DOCX]

**Supplementary material**

**Table S1. List of oligonucleotides.**

**Figure S1. Interaction of RSK and SGG.**

Lysates from non-transfected Schneider S2 cells (cont) or cells co-expressing Myc-tagged RSK and His-tagged SGG were directly analyzed by Western blot with anti-Myc and anti-His antibodies (lys) or used for immunoprecipitations (IP) with an anti-His antibody. Western Blot analysis with anti-Myc and anti-His antibodies verified an interaction of SGG with RSK.

**Figure S2. Overexpression and knock-down of SGG in clock neurons.**

(A) The *clk^856^-*Gal4 driver line was used to express *UAS-GFP* in combination with *UAS-sgg* or *UAS-sgg-RNAi* in clock neurons. Adult brains were stained at ZT20 for GFP, PER and activated SGG (pY214-SGG). In case of *sgg* overexpression, s-LN_v_ showed elevated pY214-SGG staining, whereas *sgg* knock-down caused morphological cell changes with no or residual PER and pY214-SGG signals or loss of s-LN_v_. Scale bar: 5 μm. (B) Arborization patterns of l-LN_v_ and s-LN_v_ were visualized by anti-PDF staining. Compared to wild-type (left), *sgg* knock-down resulted in loss of s-LN_v_ and their dorsal projections (arrowhead) and collapse of l-LN_v_ innervations in the medulla (arrows) with accumulation of PDF in the remaining branches. Examples for a severe (middle) and a weaker phenotype (right) are shown. Scale bar: 20 μm.

**Figure S3. Uncropped blots for Figure 1**

**Figure S4. Uncropped blots for Figure 2A-C**

**Figure S5. Uncropped blots for Figure 5B, C**
