# Supplementary figures and images for: Drosophila RSK Influences the Pace of the Circadian Clock by Negative Regulation of Protein Kinase Shaggy Activity
Source: Front Mol Neurosci. 2018 Apr 13;11:122. doi: 10.3389/fnmol.2018.00122 (PMC5908959; doi:10.3389/fnmol.2018.00122)

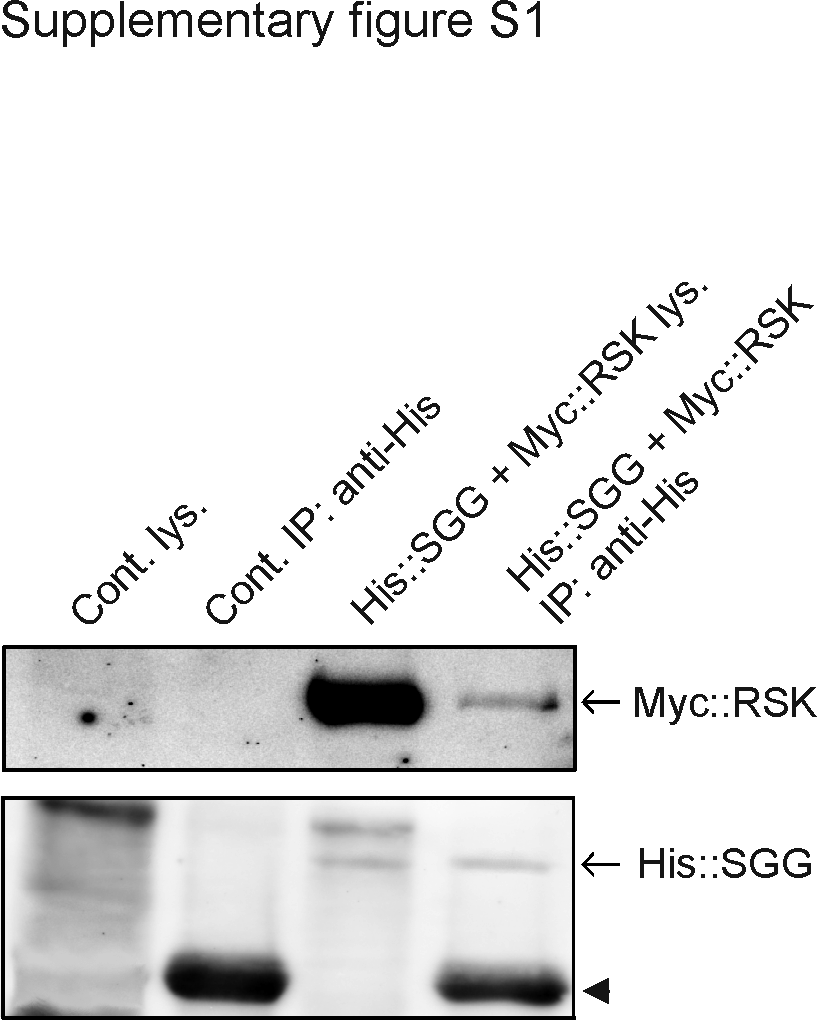

Supplement: Supplementary file 3 [file Image_1.TIF]

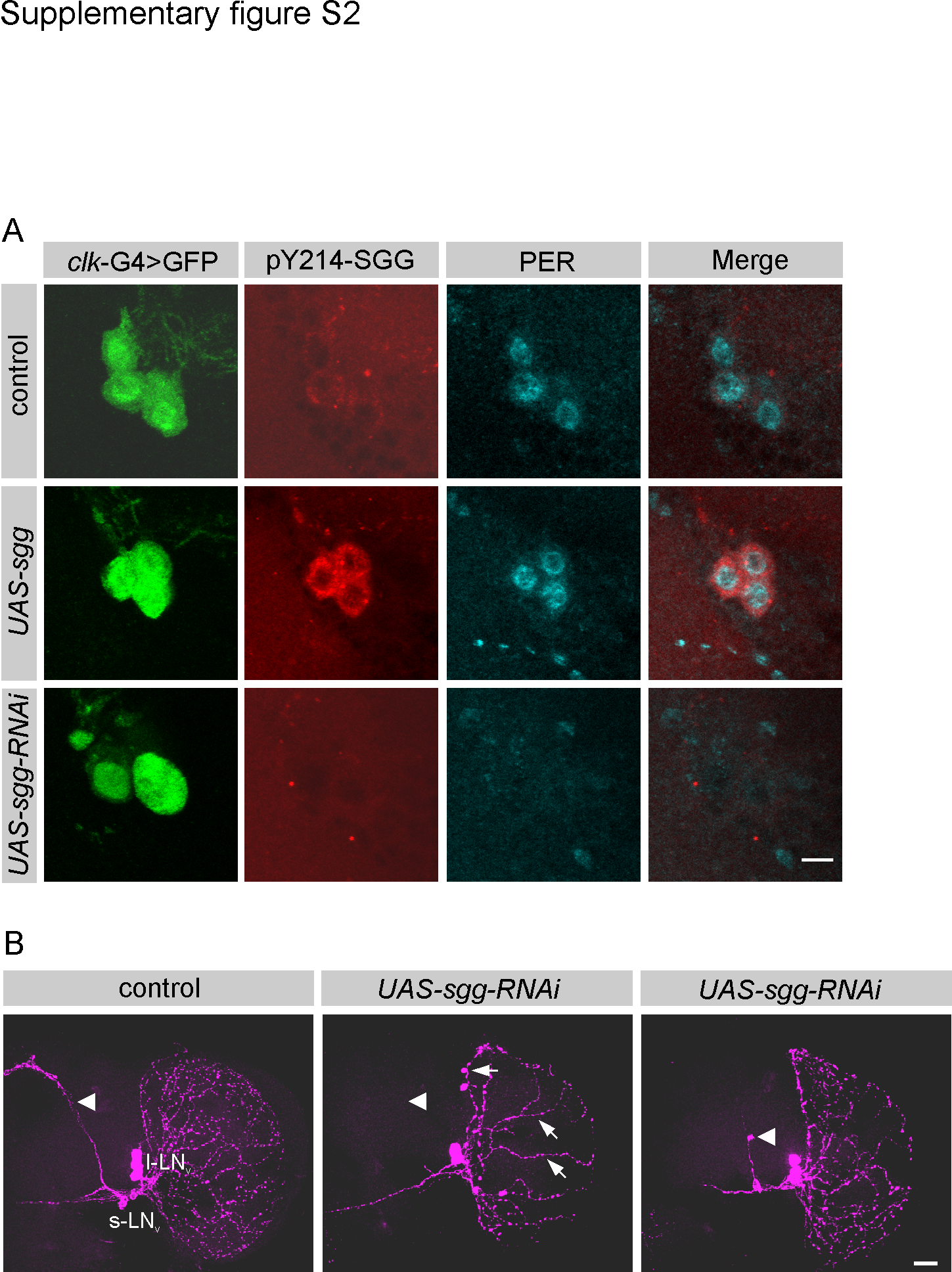

Supplement: Supplementary file 4 [file Image_2.TIF]

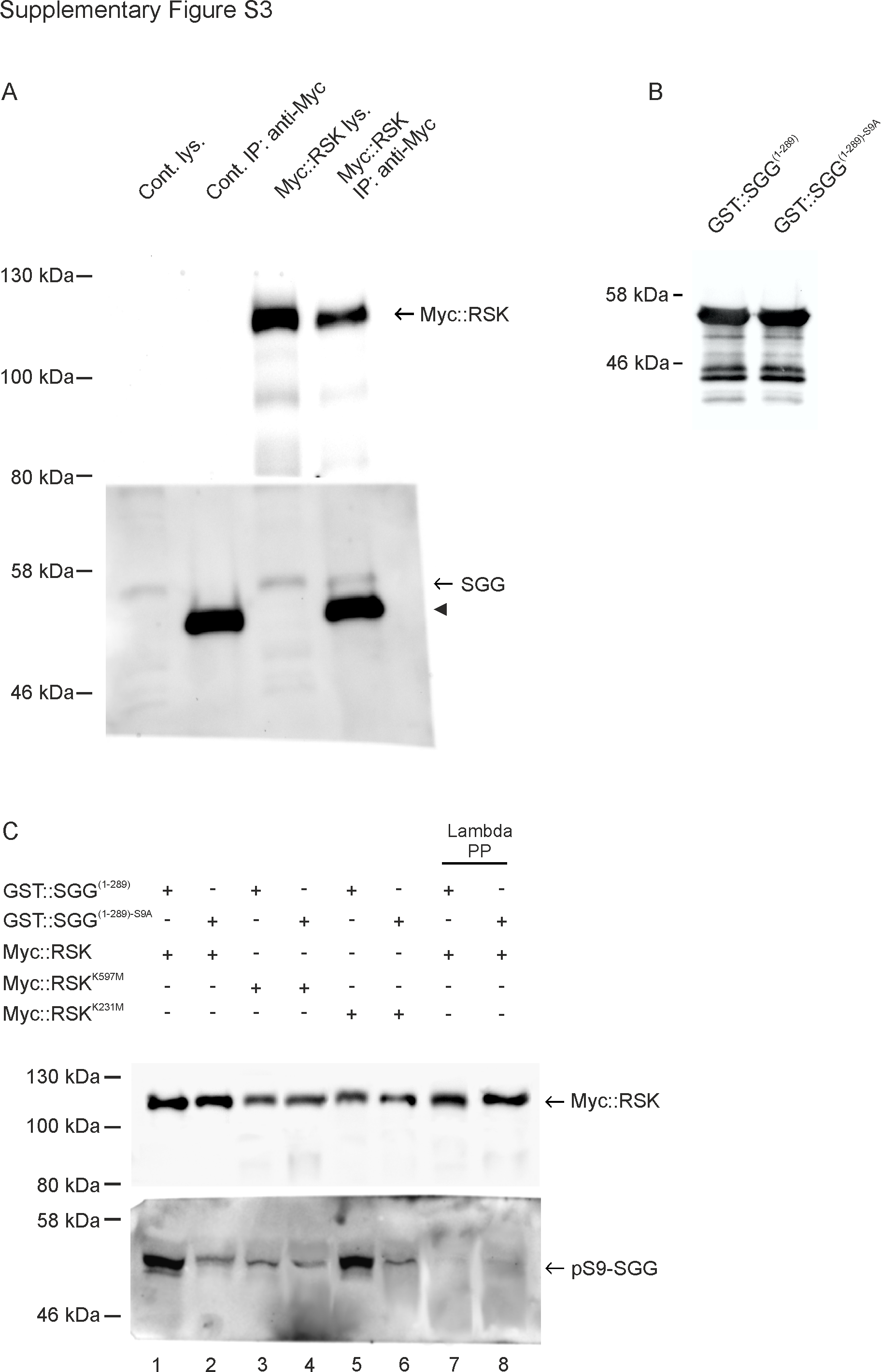

Supplement: Supplementary file 5 [file Image_3.TIF]

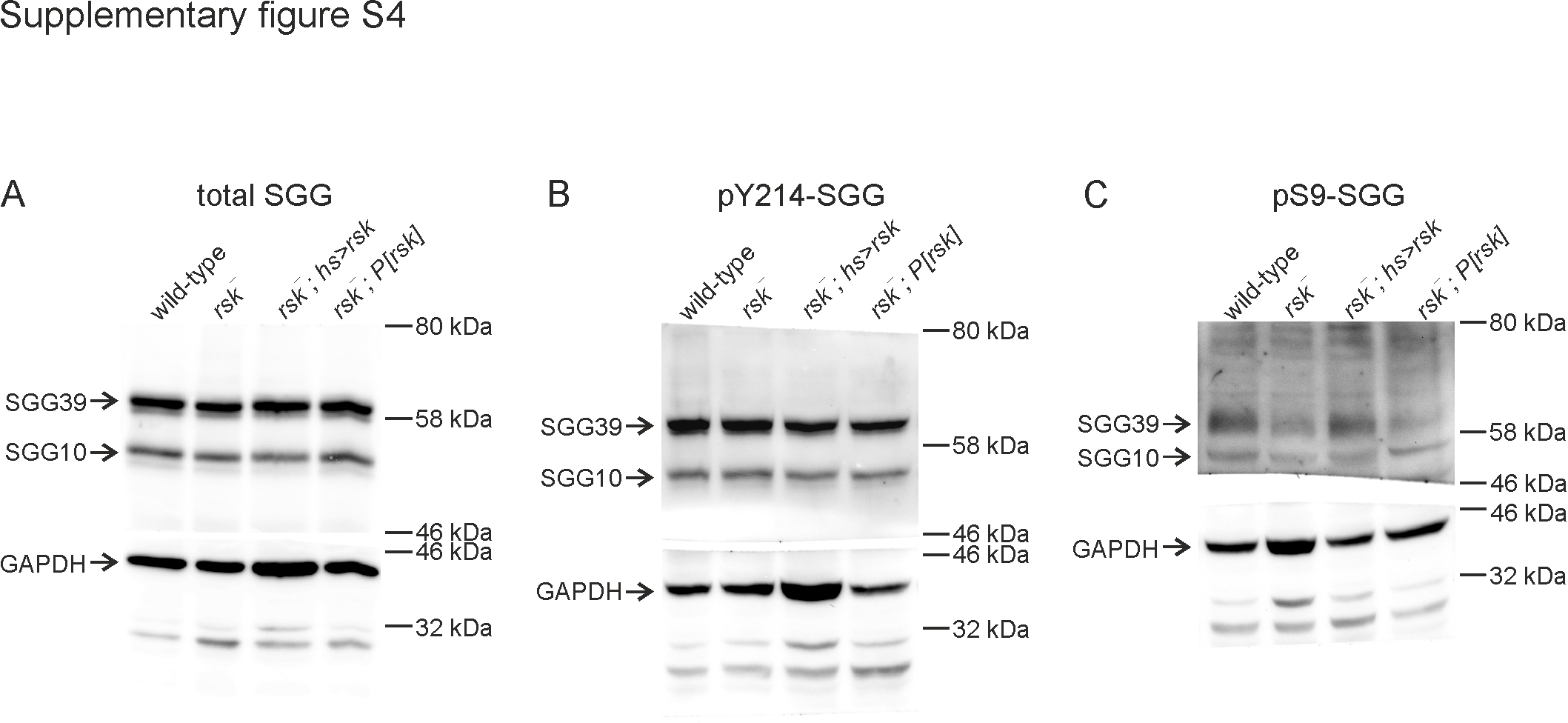

Supplement: Supplementary file 6 [file Image_4.TIF]

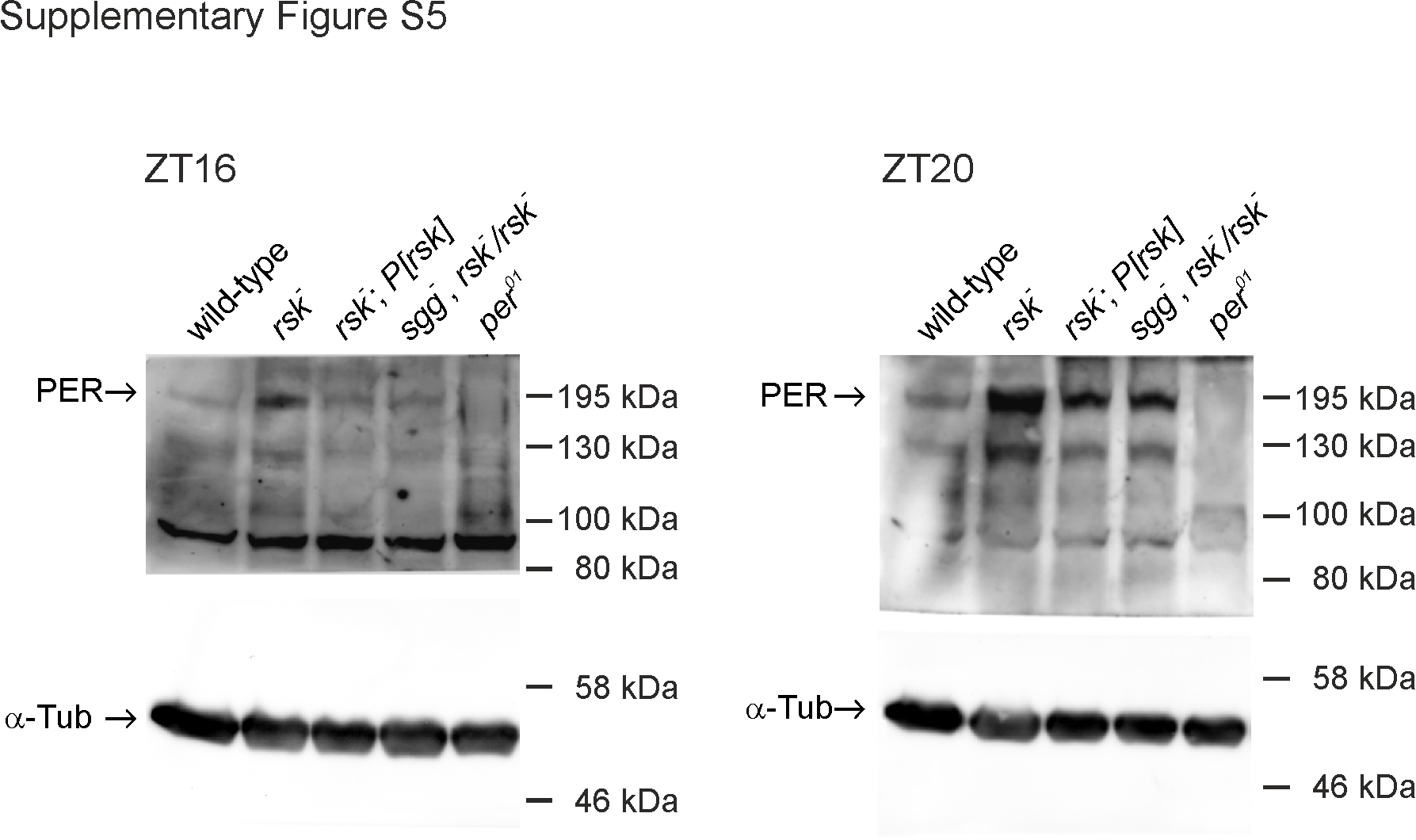

Supplement: Supplementary file 7 [file Image_5.TIF]
